# Temperament and character traits in major depressive disorder: a case control study

**DOI:** 10.1590/1516-3180.2017.0063250517

**Published:** 2017-10-02

**Authors:** Barbara Schwair Nogueira, Renerio Fraguas, Isabela Martins Benseñor, Paulo Andrade Lotufo, Andre Russowsky Brunoni

**Affiliations:** I MD, MSc, PhD. Psychologist, Hospital Universitário, Universidade de São Paulo (HU/USP), Center for Clinical and Epidemiological Research & Interdisciplinary Center for Applied Neuromodulation (Centro Interdisciplinar de Neuromodulação Aplicada, CINA), São Paulo (SP), Brazil.; II MD, PhD. Associate Professor, Department and Institute of Psychiatry, Universidade de São Paulo (USP), Center for Clinical and Epidemiological Research & Interdisciplinary Center for Applied Neuromodulation (Centro Interdisciplinar de Neuromodulação Aplicada, CINA), São Paulo (SP), Brazil.; III MD, PhD. Associate Professor, Hospital Universitário, Universidade de São Paulo (HU/USP), Center for Clinical and Epidemiological Research & Interdisciplinary Center for Applied Neuromodulation (Centro Interdisciplinar de Neuromodulação Aplicada, CINA), São Paulo (SP), Brazil.; IV MD, DrPH. Professor, Hospital Universitário, Universidade de São Paulo (HU/USP), Center for Clinical and Epidemiological Research & Interdisciplinary Center for Applied Neuromodulation (Centro Interdisciplinar de Neuromodulação Aplicada, CINA), São Paulo (SP), Brazil.; V MD. PhD. Attending Physician, Department and Institute of Psychiatry, Faculdade de Medicina, Hospital Universitário, Universidade de São Paulo (HU/USP), Center for Clinical and Epidemiological Research & Interdisciplinary Center for Applied Neuromodulation (Centro Interdisciplinar de Neuromodulação Aplicada, CINA), Service of Interdisciplinary Neuromodulation (SIN), Laboratory of Neurosciences (Laboratório de Neurociências, LIM-27), São Paulo, Brazil.

**Keywords:** Depressive disorder, major, Personality, Personality tests

## Abstract

**BACKGROUND:**

Patients with major depressive disorder (MDD) have distinct personality traits, compared with control subjects, although the role of anxiety and positive and negative affects in this finding is unclear.

**DESIGN AND SETTING:**

A case-control study enrolling 103 antidepressant-free depressed patients and 103 age and gender-matched controls was conducted at the University Hospital, University of São Paulo.

**METHODS:**

The self-reported scales of the Positive and Negative Affect Schedule (PANAS), State-Trait Anxiety Inventory (STAI) and Cloninger’s Temperament and Character Inventory (TCI) were applied. Temperament and character traits were compared between groups using multivariate and bivariate analyses of variance (MANOVA and ANOVA). The influence of anxiety and affect was further investigated using ANOVA and mediation analyses.

**RESULTS:**

Depressed patients presented higher harm avoidance and lower self-directedness scores than controls. After adjustment for anxiety trait, harm avoidance was no longer significantly different between groups. Mediation analysis revealed that the anxiety trait, but not state-anxiety or affect, fully mediated the influence of group (depressed versus control subjects) on harm avoidance.

**CONCLUSIONS:**

Our findings confirm that depressed patients present personality traits distinct from those of controls and suggest that MDD is not directly associated with harm avoidance, but that this effect is fully mediated through the anxiety trait.

## INTRODUCTION

Personality can be described as a dynamic form of organization, involving cognition, emotion, mood, impulsivity and social interactions, that modulates one’s interaction with the environment.^1^ Several models are used to study personality, from classic psychopathology and clinical psychiatry to more novel psychobiological approaches, such as the Cloninger temperament and character theory.^2^

According to Cloninger, temperament is responsible for automatic and emotional responses to environmental stimuli and encompasses four dimensions: novelty seeking (NS), consisting of behavioral activation to novel stimuli, exploratory activity, impulsivity, extravagance and disorderliness; harm avoidance (HA), a tendency towards inhibition or cessation of behaviors and avoidance of punishment and novelty; reward dependence (RD), a tendency to respond to reward and to learn to maintain rewarded behavior; and persistence (P), associated with perseverance, perfectionism, ambitiousness and eagerness of effort.

In contrast, character develops across the lifespan and is influenced by social and cultural experiences. Three dimensions are distinguished: self-directedness (SD), the ability to control and regulate behavior according to chosen goals; cooperativeness (C), associated with social acceptance and empathy; and self-transcendence (ST), associated with spirituality, altruism and identification with others.

Psychobiological models have been used in relation to several psychiatric disorders, including major depressive disorder (MDD). A recent meta-analysis^3^ showed that depressed patients present higher HA and lower SD than controls. Nonetheless, this analysis also revealed that the studies included presented low quality. For instance, several of them did not use a diagnostic interview scale, which is necessary for excluding other axis I disorders that also present altered temperament traits.^4^ Moreover, most studies enrolled patients on antidepressant treatment, which can change temperament and character inventory (TCI) scores.^5^ Furthermore, there are few studies on Brazilian populations and no study in Brazil has evaluated depressed patients using the TCI, which is important given that sociocultural factors influence character traits. Finally, most studies have not controlled for comparisons of TCI dimensions between depressed and control subjects according to anxiety and affect, which are associated with HA^6^ and character traits,^7^ respectively.

## OBJECTIVE

To fill these gaps, we aimed to compare the temperament and character traits of depressed patients with those of control subjects, in a Brazilian sample. We also explored the influence of anxiety and affect on personality traits and, particularly, on harm avoidance, which has been reported to be consistently altered in situations of depression.^3^

## METHODS

### Design

This was a case-control study in which data from depressed patients and control subjects were collected in a convenience sample. Participants were recruited between October 2013 and June 2015, at the University Hospital of the University of São Paulo, São Paulo, Brazil. Patients with depression were recruited from the Escitalopram versus Electric Current Therapy for Treating Depression Clinical Study (ELECT-TDCS),^8^ a randomized clinical trial that enrolled patients with unipolar depression. The control group was composed of age-matched (± 5 years) and gender-matched subjects recruited from among the students and staff of the University of São Paulo and through word-of-mouth.

The local and national ethics committees approved the study protocol and the participants provided informed written consent.

### Subjects

Patients aged between 18 and 75 years presenting unipolar depression were recruited. They presented a depressive episode of at least moderate severity (corresponding to a Hamilton 17-item depression rating scale (HDRS17) score ³ 17), low suicidal ideation, ability to read and understand Portuguese and at least eight years of schooling. The exclusion criteria were other axis I disorders (except anxiety disorders as a comorbidity) such as bipolar disorder (n = 9), schizophrenia (n = 6), alcohol and substance use disorders (n = 2); and any axis II (personality and developmental) disorders (n = 15). Patients who presented severe, life-threatening medical conditions and concomitant neuropsychiatric disorders such as dementia, epilepsy and stroke were also excluded (n = 14). In addition, participants needed to have been antidepressant-free for at least three weeks (five weeks for fluoxetine) at the start of the trial. Therefore, for patients using antidepressant drugs, a drug washout was performed. Benzodiazepine drugs were allowed if their use remained constant throughout the entire study and at a maximum dose of 20 mg/day (diazepam equivalent).

Controls answered a short questionnaire informing about possible psychiatric and neurological disorders and any psychotropic medication use. They were excluded if they presented important depressive symptoms.

### Assessments

The diagnoses of psychiatric disorders were confirmed by certified psychologists/psychiatrists using the Portuguese-validated version of the Mini-International Neuropsychiatric Interview (MINI).^9^ The BDI (range: 0-63; sign: positive; minimum significant score: 0-9, normal) was used to evaluate depressive symptoms.

To evaluate anxiety and affect in our sample, the Portuguese-validated versions of the State-Trait anxiety inventory (STAI; range: 0-80; sign: positive; minimum significant score: not available, N/A) and the positive and negative affective scale (PANAS; range: 10-50; sign: positive; minimum significant score: N/A)^10^ were applied. STAI evaluates state anxiety (A-state) and trait anxiety (A-trait), i.e. the presence and severity of current symptoms of anxiety and a generalized propensity to be anxious, respectively. PANAS consists of two 10-item mood scales that provide independent measurements of positive affect and negative affect (PA and NA, respectively). The Portuguese-validated, self-assessment scale of the Beck Depression Inventory (BDI)^10^ was used to evaluate depression severity. Volunteers who scored 13 or more in the BDI were not included in the control group.

Personality traits were assessed using the self-administrated Brazilian version of TCI^11^ consisting of 240 self-descriptive true/false items, assessing four temperament dimensions: NS (range 0-40; sign: positive; minimum significant score: N/A); HA (range: 0-35; sign: positive; minimum significant score: N/A); RD (range: 0-24; sign: positive; minimum significant score: N/A); P (range: 0-8; sign: positive; minimum significant score: N/A); SD (range: 0-44; sign: positive; minimum significant score: N/A); and three character dimensions: C (range: 0-42; sign: positive; minimum significant score: N/A); and ST (range: 0-33; sign: positive; minimum significant score: N/A).

### Statistical analysis

Analyses were performed in Stata 14 for Mac (Statacorp, College Station, TX, USA). A two-tailed P of 0.05 was adopted as the threshold for statistical significance. The t test (which was used for our continuous variables because they were normally distributed) and the c^2^ test (for categorical variables) were used for descriptive analysis.

Initially, we conducted multivariate analysis of variance (MANOVA) which compared all temperament and character traits (seven dependent variables) between the controls and depressed subjects. Because the MANOVA model was significant, analyses of variance (ANOVAs) were performed to assess whether each dependent variable was statistically different between the groups. Additional ANOVAs were conducted to investigate the influence of anxiety and affect on each variable.

Finally, mediation analyses were performed to further investigate the influence of anxiety and affect on mood. There were four mediation analyses, each with a different moderator variable (A-state, A-trait, PA and NA). For all analyses, group (depressed versus control subjects) was the independent variable and HA score was the dependent variable. In accordance with previous definitions,^12^ a mediator was identified when: 1) both the independent and dependent variables correlated with the mediator; and 2) no direct effect from the independent variable on the dependent variable was any longer observed when the mediation variable was included. Sobel-Goodman mediation tests were conducted (*sgmediation* package) to test whether a mediator carried the influence of an independent variable to a dependent variable.

## RESULTS

The groups were generally similar in clinical and demographic characteristics, except for a higher proportion of married people in the control group. Although the patients were not using antidepressant drugs, 19.5% of them were on low-dose benzodiazepines. The controls presented higher scores for PA and lower scores for NA, A-trait, A-scale and BDI than the depressed patients. In contrast, the patients with depression had higher scores for HA and lower scores for NS, P, SD, and C (Table 1).

**Table 1.**
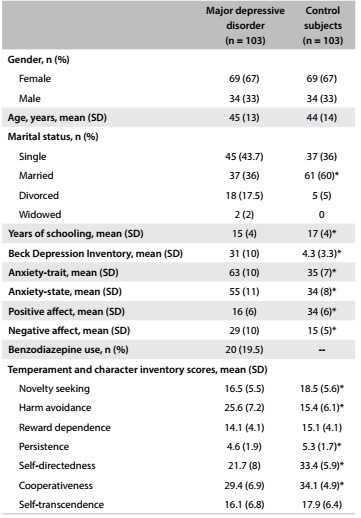
Clinical and demographic characteristics of the sample.

SD = standard deviation; n = number of subjects. *Represents variables that are significantly different between groups at P < 0.05.

The MANOVA revealed that the scores for personality traits were significantly different between the groups, with a large effect size (Pillai’s trace V = 0.498; F_7,198_ = 28; P < 0.001; h^2^_p_ = 0.498). Separate univariate ANOVAs on the dependent variables showed that all of them, except for RD and ST, were different between the groups. However, HA and NS were no longer different between the groups when A-trait was introduced as a covariate and P was not significantly different between the groups when A-state and PA were introduced as covariates (Table 2).

**Table 2.**
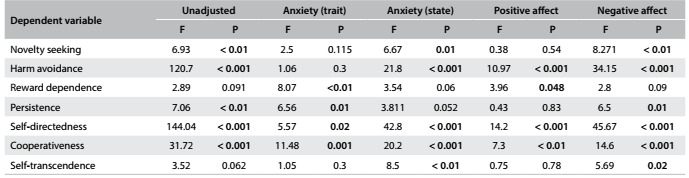
Univariate analyses on variances (ANOVAs) for personality traits.

Values in bold represent significant results at P < 0.05.

**Figure 1.**
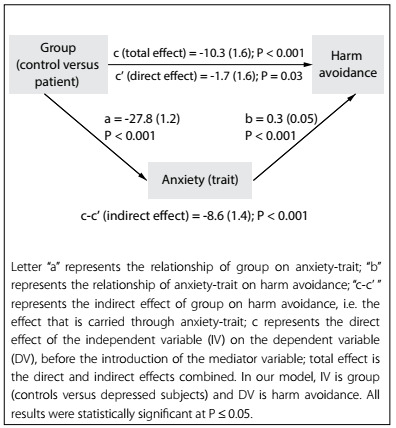
Mediating effect of anxiety-trait on harm avoidance.

Mediation analyses revealed that anxiety-trait fully mediated the relationship between the group and HA, since introduction of this variable changed the direct effect of the group on HA from significant to non-significant, and the Sobel-Goodman test revealed that the influence of the group on HA was carried, through the mediating effect of anxiety-trait (Figure 1). Moreover, anxiety-state, positive affect and negative affect were partial mediators of the influence of the group on HA, since both the direct and indirect effects in the Sobel-Goodman tests were significant (Table 3).

**Table 3.**
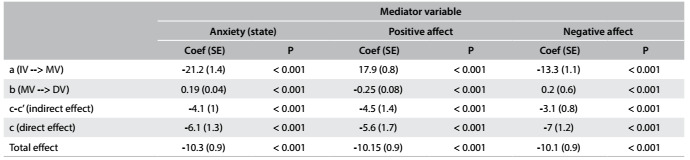
Results of the Sobel-Goodman tests evaluating the influence of group on harm avoidance according to different mediators.

a = relationship of the independent variable (IV) on the mediator variable (MV); b = relationship of the mediator variable (MV) on the dependent variable), c-c’ = represents the indirect effect of the IV on the DV, i.e., the effect that is carried through the mediator variable; c = represents the direct effect of the IV on the DV, before the introduction of the mediator variable; total effect is the direct and indirect effects combined. In our model, IV is group (controls versus depressed subjects) and DV is harm avoidance. All results were statistically significant at P ? 0.05.

## DISCUSSION

In this case-control study comparing 103 antidepressant-free depressed subjects with 103 age and gender-matched controls, the combined scores for TCI were significantly different between the groups, with a large effect size. Depressed patients presented higher HA and lower SD scores, as previously observed.^3^ This is in accordance with clinical observations showing excessive worrying, avoidant behaviors, poor goal-oriented behaviors, low confidence, and low self-esteem in cases of depression. Our patients also presented lower scores for C and P. This characteristic, along with higher scores for HA, are found in cluster C personality disorders,^13^ which are the most common personality disorders observed among depressed patients.^14^ Finally, we observed that the patients presented lower NS; however, this characteristic has not been found previously.^3^

The difference in TCI between the depressed and control participants persisted after adjustment for trait-state anxiety (STAI) and for positive or negative affect (PANAS). Nonetheless, separate analysis of each dimension revealed some distinct findings. Remarkably, the participants did not present any difference in HA scores when the comparison was adjusted for trait anxiety, which fully mediated the influence of the group on HA. Previous studies found that polymorphisms of the serotonin transporter gene were associated both with anxiety^15^ and with harm avoidance behavior.^16^ Thus, it is possible that the mediating effect observed reflected dysfunction in serotonin metabolism among participants with high HA and trait-anxiety scores. Alterations to amygdala response might also explain this finding, since chronic stress and anxiety changed amygdala responsiveness,^17^ which is involved in regulation of harm avoidance behavior.^18^

Conversely, the between-group differences in SD and C persisted even after adjustment for anxiety and mood scores. This might reflect the more stable and meta-cognitive nature of character traits, which are theoretically less correlated with anxiety and affect than temperament traits.

There were more married people in the control group. This might be explained by the greater incapacity of depressed patients in establishing or maintaining long-term relationships. Nonetheless, this issue could have partly influenced the results, since being married is associated with greater subjective wellbeing.^19^

Although the patients were antidepressant-free, 19.5% of them were on low-dose benzodiazepines. A recent study enrolling a large sample of 8,646 individuals showed that benzodiazepine use is associated with higher HA and lower SD. However, this association is larger in individuals who present benzodiazepine abuse or dependence.^20^ Nonetheless, it is possible that our results were partly driven by the small proportion of patients on benzodiazepine use.

The limitations of our study include the several comparisons performed that might have had inflated type I errors. Therefore, our findings should be interpreted as exploratory and validated in further studies. Also, due to our study design, we cannot disentangle whether the lower scores for trait variables reflected exacerbation of symptoms during depressive episodes and whether they would persist after remission from the episode. Moreover, the controls presented longer schooling than the patients, an issue that limits the strength of evidence of our findings. Finally, another important limitation is that the control group was not evaluated by psychologists or psychiatrists through a clinical interview and/or structured questionnaire.

Our findings highlight the importance of assessment of personality traits among patients with depression, which is a matter that might be overlooked by physicians and psychiatrists. Recognition that patients with depression usually present high HA and low SD is important when making clinical diagnoses and starting treatments. Moreover, we found that anxiety trait fully moderated the association between HA and avoidance. Considering that HA carries significant burden, given that it is associated with excessive worry, pessimism and avoidance behavior, clinical approaches targeted towards anxiety trait treatment might be particularly advantageous.

## CONCLUSION

Patients with depression presented a significantly different pattern of temperament and character traits, compared with control subjects, although not all dimensions differed between the groups, particularly after considering anxiety and affect. This reflects the complex and variable association of personality and depression, which involves adaptive and dynamic mental and biological systems that are interrelated at different levels. Particularly, the fully mediation effects of anxiety trait on the association between depression and harm avoidance should be further explored in longitudinal studies.
